# Association of CSF, Plasma, and Imaging Markers of Neurodegeneration With Clinical Progression in People With Subjective Cognitive Decline

**DOI:** 10.1212/WNL.0000000000200035

**Published:** 2022-03-29

**Authors:** Jarith L. Ebenau, Wiesje Pelkmans, Inge M.W. Verberk, Sander C.J. Verfaillie, Karlijn A. van den Bosch, Mardou van Leeuwenstijn, Lyduine E. Collij, Philip Scheltens, Niels D. Prins, Frederik Barkhof, Bart N.M. van Berckel, Charlotte E. Teunissen, Wiesje M. van der Flier

**Affiliations:** From the Alzheimer Center, Departments of Neurology (J.L.E., W.P., I.M.W.V., K.A.v.d.B., M.v.L., P.S., N.D.P., B.N.M.v.B., W.M.V.d.F.) and Radiology & Nuclear Medicine (S.C.J.V., L.E.C., F.B., B.N.M.v.B.), Amsterdam Neuroscience, and Neurochemistry Laboratory, Department of Clinical Chemistry (I.M.W.V., C.E.T.), and Department of Epidemiology & Biostatistics (W.M.v.d.F.), Vrije Universiteit Amsterdam, Amsterdam UMC, the Netherlands; and UCL Institutes of Neurology and Healthcare Engineering (F.B.), London, UK.

## Abstract

**Background and Objectives:**

Multiple biomarkers have been suggested to measure neurodegeneration (N) in the AT(N) framework, leading to inconsistencies between studies. We investigated the association of 5 N biomarkers with clinical progression and cognitive decline in individuals with subjective cognitive decline (SCD).

**Methods:**

We included individuals with SCD from the Amsterdam Dementia Cohort and SCIENCe project, a longitudinal cohort study (follow-up 4±3 years). We used the following N biomarkers: CSF total tau (t-tau), medial temporal atrophy visual rating on MRI, hippocampal volume (HV), serum neurofilament light (NfL), and serum glial fibrillary acidic protein (GFAP). We determined correlations between biomarkers. We assessed associations between N biomarkers and clinical progression to mild cognitive impairment or dementia (Cox regression) and Mini-Mental State Examination (MMSE) over time (linear mixed models). Models included age, sex, CSF β-amyloid (Aβ) (A), and CSF p-tau (T) as covariates, in addition to the N biomarker.

**Result:**

We included 401 individuals (61±9 years, 42% female, MMSE 28 ± 2, vascular comorbidities 8%–19%). N biomarkers were modestly to moderately correlated (range *r* −0.28 – 0.58). Serum NfL and GFAP correlated most strongly (*r* 0.58, *p* < 0.01). T-tau was strongly correlated with p-tau (*r* 0.89, *p* < 0.01), although these biomarkers supposedly represent separate biomarker groups. All N biomarkers individually predicted clinical progression, but only HV, NfL, and GFAP added predictive value beyond Aβ and p-tau (hazard ratio 1.52 [95% CI 1.11–2.09]; 1.51 [1.05–2.17]; 1.50 [1.04–2.15]). T-tau, HV, and GFAP individually predicted MMSE slope (range β −0.17 to −0.11, *p* < 0.05), but only HV remained associated beyond Aβ and p-tau (β −0.13 [SE 0.04]; *p* < 0.05).

**Discussion:**

In cognitively unimpaired older adults, correlations between different N biomarkers were only moderate, indicating they reflect different aspects of neurodegeneration and should not be used interchangeably. T-tau was strongly associated with p-tau (T), which makes it less desirable to use as a measure for N. HV, NfL, and GFAP predicted clinical progression beyond A and T. Our results do not allow to choose one most suitable biomarker for N, but illustrate the added prognostic value of N beyond A and T.

**Classification of Evidence:**

This study provides Class II evidence that HV, NfL, and GFAP predicted clinical progression beyond A and T in individuals with SCD.

In recent years, there has been a major change in the definition of Alzheimer disease (AD). Formerly, the core criteria of AD diagnosis were based on clinical symptoms.^1^ In 2018, a research framework was put forward by the National Institute on Aging–Alzheimer’s Association in which every individual is classified based on specific biomarkers in the AT(N) classification.^2^ In this framework, the term “Alzheimer disease” refers to the presence of abnormal β-amyloid (Aβ) accumulation and neurofibrillary tau tangles measured by CSF Aβ or amyloid PET (that is, “A”), and “T,” measured by CSF phosphorylated tau (p-tau) or tau PET. The AT(N) construct is independent of the cognitive stage of the individual, which makes it possible to identify AD in cognitively normal individuals. The “N” in the AT(N) classification represents neurodegeneration. Neurodegeneration can have many different causes and is not specific for AD. Therefore, neurodegenerative markers are not necessary for the diagnosis, but rather have been suggested to provide pathologic staging information and predictive value. Proposed biomarkers of N include atrophy on MRI, hypometabolism on fluorodeoxyglucose (FDG) PET, or CSF total tau (t-tau).^2^ In addition, blood-based biomarkers are now available and have been suggested as noninvasive alternative markers for N.^2-4^

Allowing different biomarkers as indicator of a biomarker group implies that they can be used interchangeably and measure the same pathologic process. For the A and T biomarker group, this assumption holds fairly well, with moderate to high agreement and relatively high correlation coefficients between markers within A and T, respectively.^5-7^ N biomarkers, however, are poorly correlated and show inadequate agreement.^6,8-11^ Furthermore, the fact that N biomarkers are suggested to provide staging information implies that individuals with a higher degree of neurodegeneration are assumed to deteriorate more quickly. However, there are few studies that directly compared different N biomarkers in their association with clinical progression or cognitive decline over time. Most are hampered by small sample sizes, and none has directly compared blood-based biomarkers with CSF and imaging biomarkers.^10,12-15^

It is difficult to determine which modality captures neurodegeneration (N) most accurately, because there is no gold standard available. However, it should capture a different process than the accumulation of Aβ (A) or fibrillary tau (T), as otherwise the addition of N would have no added value in the AT(N) classification. Furthermore, if different N biomarkers indeed capture the same process, correlations between N biomarkers should be higher than correlations between A and N or T and N biomarkers. In addition, because N provides staging information, it should have some clinical correlate. In early disease stages especially, it is important to be able to accurately predict future deterioration, for both the individuals and clinical trial recruitment, because these patients could still potentially benefit from disease-modifying therapies. Therefore, our aims were to (1) compare the different N biomarkers CSF t-tau, medial temporal atrophy (MTA) visual rating on MRI, hippocampal volume (HV), serum neurofilament light (NfL), and serum glial fibrillary acidic protein (GFAP) to each other and to markers of A and T and (2) determine their predictive value for clinical progression and cognitive decline beyond A and T in a sample of cognitively normal individuals with subjective cognitive decline (SCD).

## Methods

### Study Population

We included 401 individuals with SCD from the Amsterdam Dementia Cohort (ADC) and SCIENCe project (Subjective Cognitive Impairment Cohort).^16,17^ The SCIENCe project is a substudy of ADC and prospectively follows individuals with SCD. Individuals were referred to our memory clinic because of cognitive complaints by their general physician, a geriatrist, or a neurologist, and underwent an extensive diagnostic workup, including a physical, neurologic, and neuropsychological evaluation. In a multidisciplinary consensus meeting, all individuals received the label SCD when they performed within normal limits on a neuropsychological assessment and criteria for mild cognitive impairment (MCI), dementia, or other neurologic or psychiatric diseases that could potentially cause cognitive complaints were not met. At follow-up, diagnoses were reevaluated as SCD, MCI, AD dementia, or other types of dementia. Clinical progression was defined as progression from SCD to MCI or dementia. Inclusion criteria for the study were baseline SCD diagnosis, availability of follow-up information (≥2 diagnoses), availability of CSF, and availability of MRI or serum biomarkers within 1 year of diagnosis.

Mini-Mental State Examination (MMSE) was assessed annually and used as longitudinal measure of global cognition. Education was rated using the Dutch Verhage system.^18^

### Biomarkers

We used all biomarkers both as continuous and dichotomous measures. We used CSF Aβ (continuous and dichotomous, abnormal <813 pg/mL) or amyloid PET (dichotomous, visual assessment) as biomarker for A. When both amyloid PET and CSF Aβ were available, the PET result was used. We used CSF p-tau (abnormal >52 pg/mL) as biomarker for T. We compared 5 different N biomarkers: CSF t-tau (abnormal >375 pg/mL), MTA score (abnormal ≥1), HV, serum NfL, and serum GFAP. We used a cutoff value of ≥1 for MTA score instead of age-dependent cutoff values, to be consistent with thresholds for the other biomarkers, which are also age-independent.^19^ For HV, NfL, and GFAP, no established cutoff values were available. Because of varying rates of N+ in literature,^12,20^ we pragmatically took the 75th and 90th percentile for NfL and GFAP, and the 10th and 25th percentile for HV, which provides the reader with a range of possible effect sizes. Hence, for HV, NfL, and GFAP, we chose 2 dichotomous definitions per biomarker. The following describe the procedures used to obtain these measures.

A lumbar puncture was performed between the L3/L4, L4/L5, or L5/S1 intervertebral space to obtain CSF, which was subsequently collected in polypropylene tubes.^21^ Levels of Aβ_1-42_, tau phosphorylated threonine 181 (p-tau), and t-tau were measured using sandwich ELISAs (Innotest Aβ_1-42_, Innotest PhosphoTau_181p_, and Innotest hTAU-Ag).^22^ CSF Aβ levels were corrected for the drift that occurred over the years.^23^

For 79 individuals, amyloid PET was performed using the tracers [^18^F]florbetapir (n = 13), [^18^F]florbetaben (n = 48), [^18^F]flutemetamol (n = 7), or [^11^C]–Pittsburgh compound B (PiB) (n = 11). An IV cannula was used to administer the tracers. The following systems were used to acquire the PET scans: Gemini TF PET-CT, Ingenuity TF PET-CT, and Ingenuity PET/MRI (Philips Healthcare). For [^18^F]florbetaben^24^ and [^18^F]flutemetamol^25^ imaging, a static scanning protocol was used; for [^18^F]florbetapir^17^ and [^11^C]PiB imaging,^26^ a dynamic scanning protocol. A trained nuclear medicine physician visually rated all scans as positive or negative, according to the radiotracer specific product guidelines.

Structural MRI 3D T1-weighted images (n = 366 [89%]) were acquired as part of routine patient care from 9 different systems. The acquisition parameters are described in eAppendix 1. An experienced neuroradiologist reviewed all scans. T1-weighted images were used for visual rating of MTA (range 0–4). Scores for the left and right sides were averaged.^27^ HV was estimated using FMRIB Software Library (FSL) FIRST (v5), as described previously.^28^ The FIRST algorithm first registers the 3D T1-weighted images to the Montreal Neurologic Institute 152 template. Next, it uses a subcortical mask for segmentation based on shape models and voxel intensities to obtain HVs. HVs were normalized for head size using the V-scaling factor from SIENAX,^29^ and left and right sides were averaged. All images were visually inspected for registration or segmentation errors.

Nonfasted EDTA plasma samples (n = 296 [72%]) were obtained through venipuncture and centrifuged on average within 2 hours from collection, at 1800 *g*, 10 minutes at room temperature, before immediate storage at −80°C until analysis. Serum GFAP and NfL levels were measured using the commercially available Simoa GFAP Discovery Kit (Quanterix) and the Simoa NF-Light Advantage Kit (Quanterix) according to manufacturer's instructions and with on-board automated sample dilution.^4^ All samples were measured in duplicate with good average intra-assay % coefficient of variation.

### Standard Protocol Approvals, Registrations, and Patient Consents

The research was conducted in accordance with ethical consent by VU University and the Helsinki Declaration of 1975. For all individuals included in the study, written informed consent was available.

### Statistics

All analyses were performed in R version 4.0.3. We first used all biomarkers as continuous measures (Aβ, p-tau, t-tau, MTA, HV, NfL, and GFAP). Because the AT(N) classification is based on dichotomous variables, we repeated all analyses with dichotomized biomarkers (A, T, N_t-tau_, N_MTA_, N_HV25_, N_HV10_, N_NfL75_, N_NfL90_, N_GFAP75_, N_GFAP90_). CSF p-tau, t-tau, serum NfL, and GFAP were log transformed due to non-normality. For Cox proportional hazards models and linear mixed models, continuous predictors were transformed to *z* scores for comparability of effect sizes, and HV was inverted, so that for all variables, higher values are worse.

We first compared demographic and clinical variables between individuals who remained stable and those who progressed to MCI or dementia during follow-up, using *t* test, Mann-Whitney *U* test, and χ^2^, where appropriate. To assess correlations between biomarkers, we used Pearson correlation analysis (CSF Aβ, p-tau, and t-tau, MTA score, HV, and serum NfL and GFAP). We used partial correlation to adjust for age and sex.

We then investigated the associations between biomarkers and clinical progression using Cox proportional hazards analyses, with progression to MCI or dementia as outcome. We ran 4 different models, with a cumulative number of predictors. We first ran analyses with continuous N biomarkers as single predictors (model 1). We then added age and sex as covariates (model 2). Then we added CSF Aβ as covariate (model 3), and finally, also CSF p-tau (model 4). In models with MTA and HV, scanner type was in addition added as covariate. Separate analyses were performed for each of the N biomarkers t-tau, MTA, HV, NfL, and GFAP. Finally, for exploration purposes, we combined multiple N biomarkers in one model, entering all N biomarkers that were significantly associated with the outcome in model 4, simultaneously.

Next, we investigated the relationship between the different N biomarkers and MMSE over time using linear mixed models. We ran 4 different models with a cumulative number of covariates, similar to the models described for the Cox analyses. We first used the N biomarker, time, and N biomarker * time as predictors (model 1). Next, we added age and sex as covariates (model 2). To account for the putative modifying effect of age and sex on rate of decline, we also added the interaction terms age * time and sex * time to model 2. Then we added CSF Aβ and Aβ * time as covariates (model 3) and finally, also CSF p-tau and p-tau * time (model 4). In models with MTA and HV, scanner type was also added as covariate. We included a random intercept and random slope.

We repeated the analyses with dichotomous N biomarkers. We visualized AT(N) distributions for different N biomarkers using bar graphs. We ran Cox proportional hazards models similarly to models with continuous N biomarkers, except dichotomized N biomarkers were used as predictors, as well as dichotomized A and T biomarkers when they were added as covariates in models 3 and 4. We visualized the associations between N biomarkers and clinical progression to MCI or dementia using Kaplan Meier curves. All analyses were corrected for multiple testing using the false discovery rate (FDR). FDR-corrected *p* values <0.05 were considered significant.

### Data Availability

Data used within the article may be shared upon reasonable request.

## Results

### Baseline Demographics

The 401 individuals were on average 61 ± 9 years old, 167 (42%) were female, and 153 (39%) were *APOE* ε4 carriers (Table 1). At follow-up, 64 (16%) individuals progressed to MCI or dementia (29 [7%] to MCI, 23 [6%] to AD dementia, and 12 [3%] to non-AD dementia). Individuals who progressed to MCI or dementia were on average older, had a lower baseline MMSE score, and were more often *APOE* ε4 carriers. In addition, they had lower values for Aβ, higher values for p-tau, t-tau, MTA, NfL, and GFAP, and smaller HV.

**Table 1:**
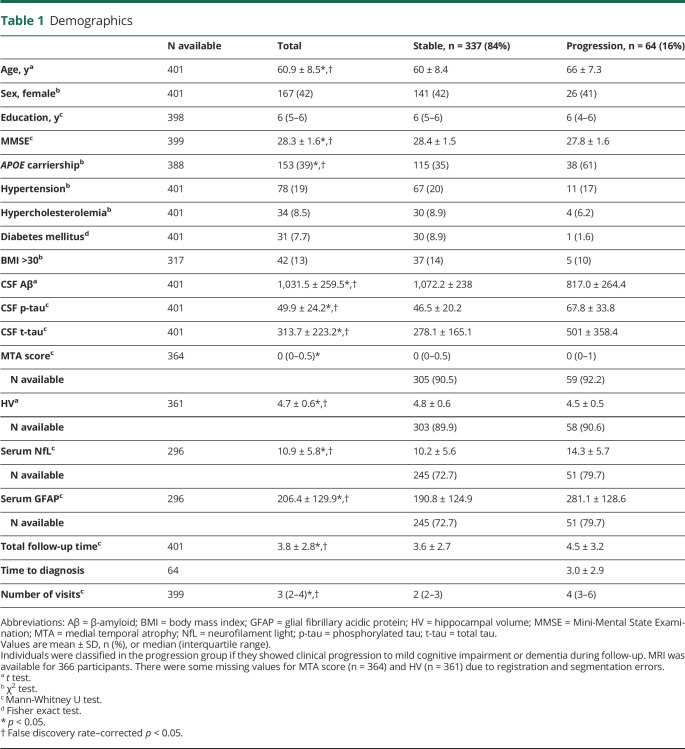
Demographics

### Correlations Between N Biomarkers

**Figure 1:**
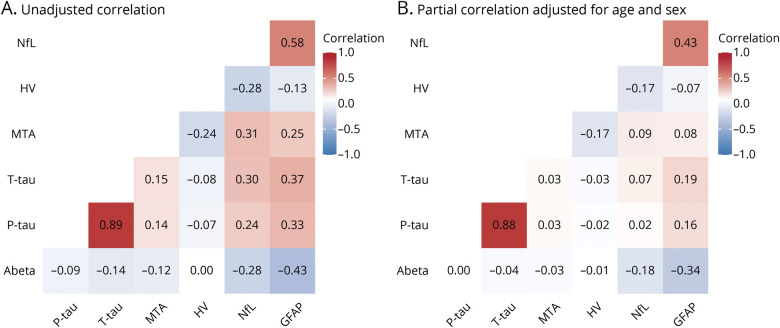
Correlations Between N Biomarkers Heatmaps showing correlations between different biomarkers. (A) Correlation coefficients (Pearson). (B) Correlation coefficients (partial correlation, adjusted for age and sex). Phosphorylated tau (p-tau), total tau (t-tau), neurofilament light (NfL), and glial fibrillary acidic protein (GFAP) were log-transformed. HV = hippocampal volume; MTA = medial temporal atrophy.

The different N biomarkers were modestly to moderately correlated (range *r* −0.28 to 0.58, Figure 1A). Serum markers NfL and GFAP correlated most strongly (*r* 0.58, *p* < 0.01). P-tau and t-tau, representing different AT(N) biomarker groups (T and N, respectively), were very strongly correlated (*r* 0.89, *p* < 0.01). Overall, the correlation coefficients between the different biomarkers for N were in a similar range as the correlation coefficients between the different biomarkers for N on the one hand and biomarkers for A and T on the other hand (*r* −0.43 to 0.33, excluding the correlation between p-tau and t-tau). After adjusting for age and sex, drastically lower coefficients were observed (Figure 1B).

### Risk of Progression to MCI or Dementia

We investigated the predictive value of the different N biomarkers using Cox proportional hazards analyses. The mean follow-up duration was 3.8 years (±2.8 years). In uncorrected models, t-tau, MTA, HV, NfL, and GFAP all predicted clinical progression to MCI or dementia (Table 2, model 1). After adding covariates in models 2 (age and sex), 3 (Aβ, age, and sex) and 4 (Aβ, p-tau, age, and sex), hazard ratios (HRs) were attenuated. Model 4 showed that HV, NfL, and GFAP added predictive value to Aβ and p-tau. T-tau also predicted MCI or dementia in models 1 to 3, but was not entered in model 4 due to collinearity between t-tau and p-tau. In an additional explorative analysis, we added the 3 N markers HV, NfL, and GFAP simultaneously in a model in addition to Aβ and p-tau, because these biomarkers added predictive value in model 4. In this model, only HV remained significantly associated with clinical progression to MCI or dementia (HR 1.45 [SE 1.01–2.09]). The associations for NfL (0.94 [0.56–1.59]) and GFAP (1.40 [0.86–2.29]) were attenuated (n = 258 due to varying availability rates for N biomarkers).

**Table 2:**
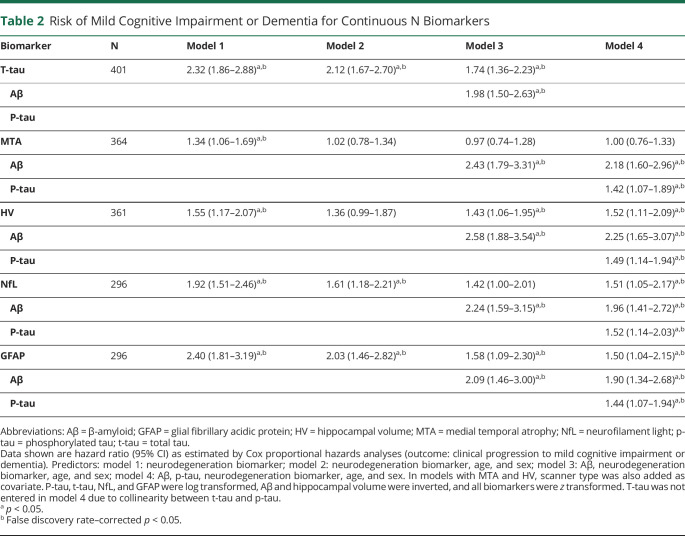
Risk of Mild Cognitive Impairment or Dementia for Continuous N Biomarkers

Results of the analyses for complete cases only (n = 256) were overall similar, although not all associations survived FDR correction (eTable 1, links.lww.com/WNL/B780).

### Cognitive Decline Over Time

We estimated change in MMSE over time using linear mixed models. In total, 1196 MMSE scores of 399 participants were available, with missing values for 2 individuals (334 ≥ 2 visits; range 1–17, median 3 visits). No associations between any N biomarkers and baseline MMSE scores were observed in our sample of cognitively normal elderly. Table 3 shows the results for the interaction between the N biomarkers and time, which reflects the effect of each of the N biomarkers on MMSE slope. In both uncorrected models (model 1) and models corrected for age and sex (model 2), t-tau, HV, and GFAP predicted MMSE slope. T-tau and HV also added predictive value to Aβ (model 3), but only HV added predictive value beyond Aβ and p-tau (model 4). Results were similar for analyses with complete cases (n = 256, eTable 2).

**Table 3:**
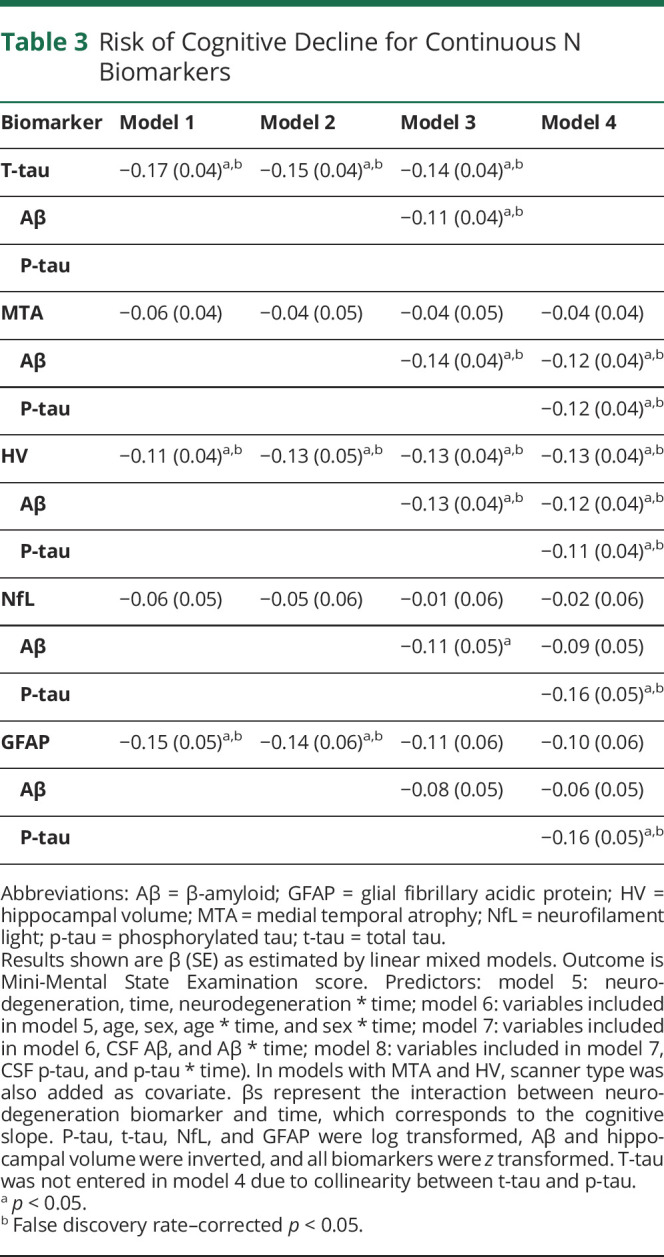
Risk of Cognitive Decline for Continuous N Biomarkers

### Dichotomous N Biomarkers

**Figure 2:**
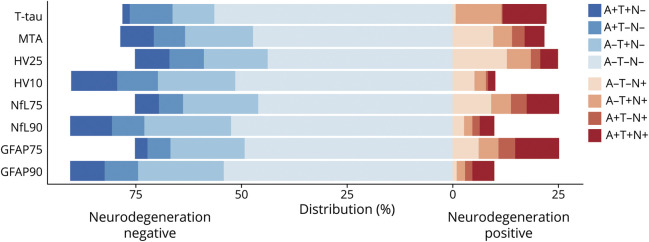
Distribution of AT(N) Profiles According to Different Definitions of Neurodegeneration Distribution of AT(N) profiles for different definitions of neurodegeneration. GFAP 75 = glial fibrillary acidic protein, threshold 75th percentile; GFAP 90 = glial fibrillary acidic protein, threshold 90th percentile; HV 10 = hippocampal volume, threshold 10th percentile; HV 25 = hippocampal volume, threshold 25th percentile; MTA = medial temporal atrophy; NfL 75 = neurofilament light, threshold 75th percentile; NfL 90 = neurofilament light, threshold 90th percentile; t-tau = total tau.

The proportion of N+ individuals, and hence the distribution of AT(N) categories, strongly depended on the definition of N (Figure 2). Proportions of N+ varied between 10% (N_HV10_, N_NfL90_, N_GFAP90_) and 25% (N_HV25_, N_NfL75_, N_GFAP75_). For N_t-tau_ and N_MTA_, proportions of N+ were about 22%. N+ was more common in A– compared to A+ individuals for N_MTA_ or N_HV_, and more common in A+ compared to A– individuals for N_GFAP_. For N_NfL_ and N_t-tau_, frequencies of N+ were similar between A+ and A–.

**Figure 3:**
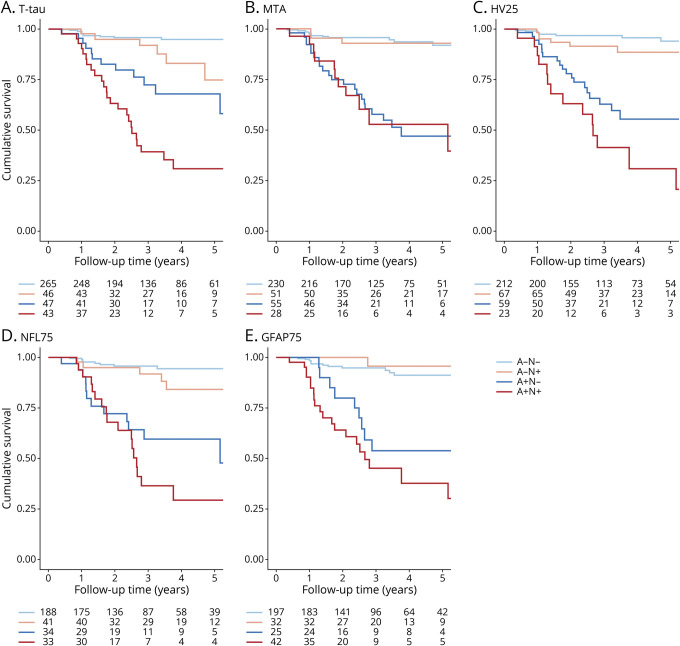
Kaplan-Meier Curves Visualizing Clinical Progression Within a Classification Kaplan-Meier curves visualizing clinical progression to mild cognitive impairment or dementia for different definitions of neurodegeneration (A, total tau [t-tau]; B, medial temporal atrophy [MTA]; C, hippocampal volume, threshold 25th percentile [HV 25]; D, neurofilament light, threshold 75th percentile [NFL 75]; E, glial fibrillary acidic protein, threshold 75th percentile [GFAP 75]). Survival is visualized by constructing a 4-level variable of dichotomous amyloid and neurodegeneration status (A–N–, A–N+, A+N–, A+N+).

Cox proportional hazards analyses using dichotomous N biomarkers to predict clinical progression to MCI or dementia provided overall similar results to analyses with continuous biomarkers for models 1 and 2 (Table 4). However, only N_t-tau_ and N_HV25_ added predictive value to A, and only N_HV25_ added value beyond A and T. Figure 3 visualizes the combined effect of A and N status for each N on risk of clinical progression in 4-level variables (A–N–, A-N+, A+N–, A+N+).

**Table 4:**
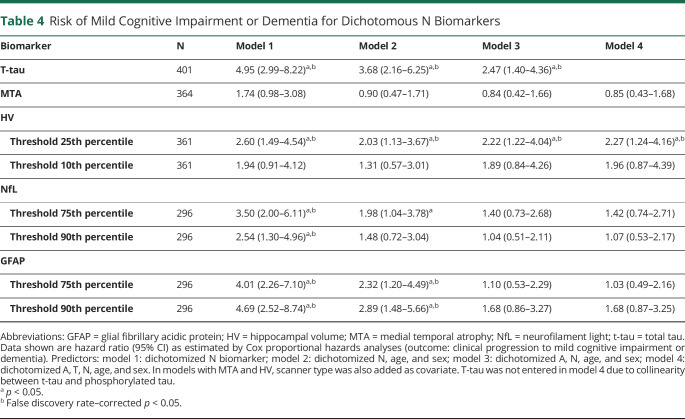
Risk of Mild Cognitive Impairment or Dementia for Dichotomous N Biomarkers

### Classification of Evidence

This study provides Class II evidence that HV, NfL, and GFAP predicted clinical progression beyond A and T in cognitively unimpaired elderly individuals with SCD.

## Discussion

In a sample of cognitively normal individuals with SCD, we found modest to moderate correlations and low concordance among the N biomarkers t-tau, MTA, HV, NfL, and GFAP. N biomarkers HV, NfL, and GFAP each predicted clinical progression, and had predictive value in addition to Aβ and p-tau. Therefore, we recommend HV, NfL, or GFAP as biomarkers for N. The tight correlation between t-tau and p-tau precludes the use of the former as a marker of a different biomarker category than the latter.

We extend former observations that different markers of N are not necessarily closely correlated. The low correlation between N biomarkers likely contributes to the often discordant biomarker results in the AT(N) classification.^6,9,12,15^ We add blood-based biomarkers to the comparison, showing similarly modest associations with the N biomarkers in other modalities, and also similarly strong associations with clinically relevant outcomes. Although at a population level, the overall qualitative pattern of biomarker frequencies remains rather stable regardless of the type of biomarkers used,^9^ it becomes problematic when researchers and clinicians treat the different N biomarkers as if they were identical. For prediction modeling at the individual patient level, the prognosis for an individual will vary considerably depending on the choice of N biomarker. The choice of N biomarker will also have an effect on the design of therapeutic trials, as well as the potential implementation of the AT(N) classification in the clinic. Studies investigating the AT(N) classification that use different definitions of their biomarkers cannot be directly compared.

We found low to modest correlations and low concordance between different N biomarkers, which is largely in line with the literature.^6,10,12,30,31^ One possible explanation for this is that although all N biomarkers capture a certain aspect of neurodegeneration, the underlying biological processes that lead to specific N biomarker abnormalities are far from identical. T-tau and NfL reflect the severity of neuroaxonal injury, atrophy on MRI reflects loss of the neuropil, and GFAP reflects astrocyte activity.^2,32-34^ Literature suggests these processes all have a different longitudinal trajectory; for example, NfL and t-tau abnormality likely precede HV abnormality and t-tau eventually reaches a plateau.^35-38^ This means correlations between N biomarkers of different processes are probably dependent on disease stage. However, MTA and HV were also poorly correlated, which is remarkable considering both HV and MTA aim to measure a similar process. We found a correlation coefficient of −0.24, which is relatively low and slightly lower than coefficients found in literature (range *r* −0.27 to −0.54).^39-41^ This low correlation could be due to the fact that the MTA score is partly influenced by the volume of the surrounding CSF spaces, which means it reflects hippocampal atrophy as well as global and subcortical atrophy.^42^ Furthermore, being cognitively normal, most individuals in our sample had an MTA score of 0, which reflects that the variability for this measure is probably too small to be a meaningful N biomarker in such a very early sample. In addition, the correlation coefficients between N biomarkers were in a similar range as the correlation coefficients between N biomarkers on the one hand and A and T biomarkers on the other hand. This is in line with another study that found moderate correlations between biomarkers of different pathophysiologic categories.^6^ This implies that the underlying neurodegeneration processes are almost as different from each other as they are different from processes underlying the A and T biomarker category. Overall, the low correlation coefficients illustrate that N biomarkers cannot be used interchangeably in the AT(N) classification.

We found that HV, NfL, and GFAP predicted clinical progression, and HV predicted MMSE slope, beyond Aβ and p-tau. Former studies that investigated the AT(N) classification often used only one biomarker for A, T, and N, respectively, and showed that overall, the AT(N) classification was associated with clinical progression and cognitive decline.^20,43-47^ From these studies, the predictive value per individual biomarker cannot be discerned and thus cannot be used to choose the optimal N biomarker. Literature regarding the comparison between different N biomarkers is more scarce. There is, however, some support that HV is associated with cognitive decline and progression more strongly than t-tau.^13-15^ Although in our study we found t-tau as individual biomarker also predicted clinical progression and cognitive decline, the high correlation with p-tau hampers the addition of t-tau to a model with Aβ and p-tau, making it a less desirable biomarker to use in the AT(N) classification. NfL and GFAP have both been shown to be related to baseline cognition, cognitive decline, and clinical progression as individual predictors, but have not yet been studied extensively in comparison to other N biomarkers.^3,48-50^ In a former study, we found GFAP was more strongly related to clinical progression and cognitive decline than NfL, which is in line with our current study.^4^ We found both GFAP and NfL predicted clinical progression beyond Aβ and p-tau, but NfL was not associated with MMSE decline. A potential explanation for this difference in association is that NfL is a better marker for monitoring disease progression while its value does not lie in predicting future cognitive decline.^4^ Differences could also be related to the fact that clinical progression to MCI or dementia is a binary outcome measure, while MMSE decline is a continuous measure with possibly a higher degree of measurement variation. Clinical progression might be a more sensitive measure with more clinical relevance. In contrast to NfL, GFAP was associated with MMSE decline, although associations were attenuated when in addition adjusting for Aβ or p-tau. Of all N biomarkers we used, GFAP was associated most strongly with Aβ, which could explain the attenuated estimates when Aβ was added as covariate. MTA was not associated with clinical progression after correcting for covariates or with MMSE decline. Although we previously showed a dose–response pattern with MTA as N,^20^ the small variability in MTA within cognitively normal individuals makes it too crude a measure to accurately predict decline. Overall, we show there is room for improved prediction beyond Aβ and p-tau, using HV, NfL, and GFAP as N biomarkers.

Limitations of the current study include that the list of N biomarkers examined is not exhaustive. For example, FDG-PET or other MRI atrophy measures have also been suggested as suitable N markers. Although the list of putative N biomarkers is long, we chose to use a variety of N biomarkers obtained by 3 different modalities that are widely used in literature, which makes our study relevant to the field. Another limitation is that the sample sizes somewhat differed for each N biomarker. This might have led to differences in outcome. However, when we repeated the analyses in the sample with complete data, results were similar, indicating their robustness (eTables 1 and 2, links.lww.com/WNL/B780). Furthermore, our sample consisted of individuals with SCD presenting at a memory clinic, and the results might not be directly translatable to a community-based setting or to other disease stages. Nonetheless, individuals with SCD can be considered an especially clinically relevant group that might particularly benefit from the AT(N) classification system to grade their degree of underlying pathology. These are individuals who present to a memory clinic because of worries about their cognition, and for this group AT(N) prediction modeling can make a relevant contribution. Another limitation is the lack of optimal cutoff values for HV, NfL, and GFAP. Instead, we pragmatically used cutoff values obtaining a 10% and 25% N positivity rate to provide a range of the true effect sizes. In addition, we used continuous N biomarkers in all models. However, different cutoff values would probably have resulted in slightly different results. Lastly, we had a mean follow-up duration of 3.8 years and our sample had a relatively young age. Together, this could explain the low percentage of individuals with clinical progression to MCI or dementia, which limits the power to detect associations with N biomarkers. Furthermore, MMSE has a ceiling effect in cognitively normal individuals and our relatively short follow-up time may have hampered the finding of associations. Because all N biomarkers reflect different aspects of neurodegeneration, they could also have different associations with cognitive tests measuring specific cognitive domains. It would be interesting to investigate associations with other neuropsychological tests, but that is beyond the scope of this study because our aim was to assess the association between N biomarkers and disease progression in general. Strengths include the relatively large sample size of this well-defined cohort.

Correlations between different N biomarkers were low in this sample of cognitively normal individuals, indicating they may not reflect the same underlying pathology. T-tau was strongly associated with p-tau, and thereby disqualified as measure for N in this context. Our results show that HV, NfL, and GFAP predicted clinical progression, and have added value beyond Aβ and p-tau. However, our results do not reveal a single most suitable biomarker for N.

## Glossary

Aβ: β-amyloid
AD: Alzheimer disease
ADC: Amsterdam Dementia Cohort
FDG: fluorodeoxyglucose
FDR: false discovery rate
GFAP: glial fibrillary acidic protein
HR: hazard ratio
HV: hippocampal volume
MCI: mild cognitive impairment
MMSE: Mini-Mental State Examination
MTA: medial temporal atrophy
NfL: neurofilament light
p-tau: phosphorylated tau
PiB: Pittsburgh compound B
SCD: subjective cognitive decline
SCIENCe: Subjective Cognitive Impairment Cohort
t-tau: total tau
